# The difference in dissipation of clomazone and metazachlor in soil under field and laboratory conditions and their uptake by plants

**DOI:** 10.1038/s41598-020-60720-0

**Published:** 2020-02-28

**Authors:** Ewa Szpyrka, Magdalena Słowik-Borowiec, Paulina Książek, Aneta Zwolak, Magdalena Podbielska

**Affiliations:** University of Rzeszow, Institute of Biology and Biotechnology, 1 Pigoń St., 35-310 Rzeszów, Poland

**Keywords:** Plant sciences, Environmental monitoring

## Abstract

The study concerned dissipation of metazachlor and clomazone, herbicides widely used in rapeseed (*Brassica napus* L. subsp. *napus*) protection, applied to the clay soil under field and laboratory conditions. Furthermore, the uptake of these pesticide from soil by rapeseed plants was investigated under field conditions. An additional aim of this work was to modify the QuEChERS method for the determination of metazachlor and clomazone in the plant material. Analytical procedures for metazachlor and clomazone qualification and quantification in rapeseed plants and soil were developed, using gas chromatography with an micro electron capture detector (GC-μECD) and a mass detector (GC-MS/MS QqQ) as confirmation. Dissipation kinetics of herbicide residues in soil were described as first-order equations. The analytical performance was very satisfactory and confirmed that the methods meet the requirements of the European Commission. In the conducted field experiments it was found that dissipation of clomazone and metazachlor in clay soil follows first-order kinetics (R^2^ between 0.964 and 0.978), and half-lives were 9.5 days and 10.2 days for clomazone and metazachlor, respectively. Under laboratory conditions, dissipation of clomazone and metazachlor in soil also follows first-order kinetics (R^2^ between 0.937 and 0.938), and half-lives were 8.8 days and 5.7 days for clomazone and metazachlor, respectively. Residues of both herbicides in rape plants 22 days after application of herbicides were below the maximum residue levels for Brassica plants. Metazachlor and clomazone dissipate very fast in clay soil and their uptake by rape plants is very low.

## Introduction

The constantly growing demand for food requires the use of modern methods and technologies in agricultural production, including the use of plant protection products. As it is widely known, there is some evidence of advantageous effects of pesticides, such as an increase in the quantity and quality of crops^1^, but, at the same time, the intensification of agriculture, including the use of these inherently toxic and highly persistent substances, can lead to environmental contamination, as well as consumer exposure that may be harmful to their health^2–4^. Because this problem raises serious social concerns, there are many governmental and international institutions that are responsible for establishing laws in this area. Codex Alimentarius^5^ or The Environmental Protection Agency^6^ establish maximum residue levels (MRLs) for pesticides in food, while in the European Union levels of pesticides are controlled in accordance with Regulation 396/2005^7^ and Regulation 149/2008^8^.

Indisputably, soil is an important agricultural resource able to produce, distribute and store matter and chemicals^9^. Soil may be exposed to both industrial and agricultural pollutions, including pesticides. These substances can be introduced into the soil in various ways, e.g. by direct application, accidental spillage, runoff from plants or by the inclusion of plant materials contaminated with pesticides. Once in the soil, pesticides can undergo one or more of the following processes: adsorption, leaching, degradation, evaporation, chemical and microbiological changes, or movement into the soil profile^10^. The use of herbicides in crop fields allows limiting crop losses related to the competitiveness of weeds. Herbicides penetrate plants through their leaves and/or roots, and then they are distributed through a vascular system, disrupting plant’s vital processes. The level of herbicide residues in plants depends on the ability to collect and metabolize the active substance by a given species. Weather conditions and a dose of a formulation also play an important role. Whereas, the degradation rate and/or the persistence of herbicides in the soil depend mainly on an active substance type, and on its physical and chemical properties, environmental conditions (soil type, temperature, humidity, pH), and microorganisms (bacteria and fungi)^11,12^. Biodegradation of herbicides is faster in soils rich in microbes than in barren soils. For the complete inactivation of an herbicide, an interaction of various types of microorganisms is usually needed. Microbial degradation occurs fastest in surface soil layers, where the intensity of biological life is the highest. When intensive rainfall occurs after spraying, the active substance may move faster into the soil profile, where the prevailing conditions are less favorable for microflora and the microbial degradation is slower. The number and activity of soil microorganisms, and thus the rate of microbial degradation, is strongly influenced by soil temperature and humidity. Generally, as the temperature and humidity increase, the microbial degradation process accelerates. Periods of drought or low temperatures inhibit the growth of microorganisms, resulting in slowing down the decomposition of herbicides^11^.

Because herbicide treatments can be a source of pollution of plant products and of the soil environment, it is important to monitor these substances by multiresidue methods, facilitating an accurate and reliable measurement of their concentration levels, as well as a risk assessment^10,13^.

The literature describes many analytical methods available for the determination of pesticide residues in plant material and soil samples. To a large extent, these methods are based on traditional approaches: soxhlet extraction^14–16^, solid phase extraction^17,18^, pressurised liquid extraction^19,20^, matrix solid-phase dispersion^21,22^, solid-phase microextraction^23,24^, or dispersive liquid-liquid microextraction^25^. Currently, the QuEChERS procedure^26^ is one of the most commonly used analytical methods for the determination of pesticides residues, and frequently this procedure is modified in terms of amounts and types of reagents, as well as available equipment^27–30^.

Information on the presence of pesticide residues in rapeseed plants or the dissipation of these substances is very limited, and only a few literature references concern the content of pesticides in oilseed rape or oil seeds^31–34^. For this reason, focusing our research on this area seems to be justified.

Herbicides containing clomazone and metazachlor as active substances are very popular and are recommended for simultaneous application in rapeseed crops. In the literature, the availability of information about the dissipation of these two herbicides in soil is limited, and the information about uptake of herbicide residues by rapeseed plants is scarce; therefore, these studies can be considered as a novel approach. The aim of the study was to analyze dissipation kinetics of clomazone and metazachlor under field and laboratory conditions, together with the uptake of residues of these herbicides by rapeseed plants. The additional aim was to develop and validate a method for the determination of metazachlor and clomazone in the plant material, as well as validation of a method for the analysis of these substances in the soil.

## Results

### Soil parameters

The soil was characterized by following parameters: soil type – clay; the granulometric composition: 2.37% of 1.0–0.1 mm fraction, 54.34% of 0.1–0.02 mm fraction, and 43.29% of <0.02 mm fraction; soil pH 6.9; assimilable phosphorus –20.1 mg P_2_O_5_/100 g; total carbon – 1.02%; organic carbon – 0.87%; humus content – 1.5%; total nitrogen – 0.12%; phosphorus content – 850.20 mg/kg, magnesium – 0.28%, potassium – 0.49%, and calcium – 0.30%.

### Validation of a method for ananalysis of clomazone and metazachlor in soil

The average recovery in soil samples was determined for two fortification levels: 1 mg/kg for both substances, and 0.01 mg/kg for clomazone, and 0.005 mg/kg for metazachlor. Precision was calculated from the recovery experiments and it was expressed as RSD at each spiking level. For all pesticides, a percentage range of mean recovery and RSD was acceptable and amounted to 88–112% and 7–13%, respectively. The results obtained are presented in Supplementary Table S1.

Linearity of calibration curves in the “blank” matrix was studied over the concentration range between 0.01–2.88 μg/mL for clomazone and 0.005–2.12 μg/mL for metazachlor, by gas chromatography with a micro electron capture detector (GC-µECD) analysis with satisfactory coefficients of determination (R^2^) ≥0.99.

The limit of quantification (LOQ) for each pesticide were set at the lowest spiking concentrations (0.01 mg/kg for clomazone and 0.005 mg/kg for metazachlor), for which validation criteria, in the term of recovery and precision, were fulfilled^35^.

The expanded uncertainty was calculated as twice the value of the uncertainty (k = 2, confidence level 95%) and ranged between 16% and 27%, with an average of 21%.

### Modification and validation of clomazone and metazachlor method for analysis of rapeseed

A modified and validated QuEChERS method was used to analyze metazachlor and clomazone residues in rapeseed plants. The method for preparing rapeseed samples was modified versus the standardized method. The changes included reduction of the plant material weight to 5 g, addition of 10 mL of water, and a changed sorbent used for clean-up, i.e., we used florisil and PSA. Florisil sorbent is not listed in the QuEChERS method, but is suitable for chlorophyll removal, while PSA is recommended for clean-up of Brassica samples^36,37^. To check the effectiveness of the rapeseed extracts’ purification, the validation of plant samples enriched with the tested herbicidal active substances was conducted.

The fortification levels were 0.010 and 1.000 mg/kg for clomazone and 0.005 and 1.000 mg/kg for metazachlor. Recoveries were determined in five repetitions per each of the two relevant two spiking levels (Supplementary Table S2). Precision was calculated from the recovery experiments, and it was expressed as RSD at each spiking level. For both tasted sorbents, average recovery values obtained for metazachlor and clomazone in rape ranged between 86% and 109%, with RSD values below 12%. Details are presented in Supplementary Table S2. When compared with the validation criteria for the EU method according to SANTE^35^, where the average recovery between 70 and 120%, and RSD lower than or equal to 20% is recommended, satisfactory validation results were obtained for both analyzed substances.

To assess the selectivity of the method, chromatograms of the prepared samples cleaned with the analyzed sorbents were evaluated. Comparing both chromatograms of cleaned extracts, it can be concluded that samples cleaned with florisil contained less peaks from co-extracted substances (Supplementary Fig. S1).

Linearity was studied by analyzing matrix-matched standards in “blank” matrix at six concentration levels. The calibration curves were linear in ranges of 0.01–2.88 μg/mL for clomazone and 0.005–2.12 μg/mL for metazachlor, with coefficients of determination (R^2^) ≥0.99 for both analytes (Supplementary Table S2).

The lowest spiking level corresponded to the LOQ, ensuring LOQ values corresponding to the MRLs (for Brassica plants, MRL is 0.02 mg/kg for metazachlor and 0.01 mg/kg for clomazone)^38^.

Method uncertainty (U) for metazachlor and clomazone was calculated following the “top-down” approach using overall recovery and precision data^29,39^, reaching 6–26% (16% on average) for metazachlor and 7–16% (11% on average) for clomazone (coverage factor k = 2, confidence level of 95%). These values are much below the limit value of 50%, recommended by European Union guidelines^35^.

### Dissipation of herbicides in soil and substance uptake by rapeseed

Samples of soils from field were collected 1, 5, 13, 22 and 34 days after herbicides application, while samples of rapeseed whole plants were collected after emergence, 22 and 34 days after herbicides application.

**Figure 1:**
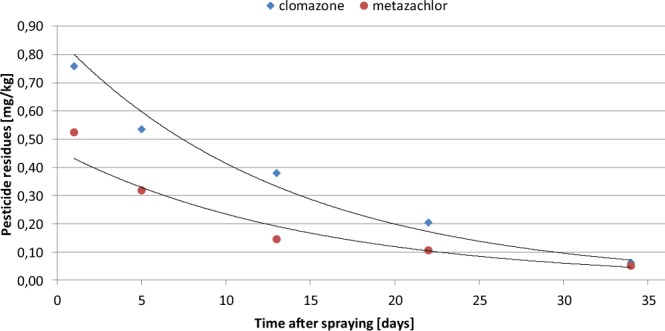
Dissipation of clomazone and metazachlor in the soil under field condition.

**Figure 2:**
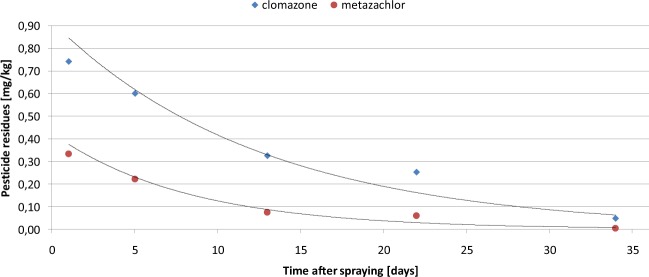
Dissipation of clomazone and metazachlor in the soil under laboratory condition.

The laboratory tests were conducted simultaneously with the field experiment. Soil samples collected from field one day after herbicide application were stored under laboratory condition and analyzed at the same times as samples from the rapeseed field. All samples were analyzed using the validated methods described previously. The overall results of the soil and rapeseed analyses are presented in Table 1 and Figs. 1 and 2.

**Table 1:** Average residue concentrations of clomazone and metazachlor in soil and rapeseed plants after spraying at recommended doses: clomazone – 0.2 L/ha; metazachlor – 1 L/ha; LOQ – limit of quantification.

Time (days)	Residue level (±RSD) (mg/kg)		
	Clomazone	Metazachlor	
***Soil – field condition***			
1	0.758 ± 0.025		0.524 ± 0.455
5	0.536 ± 0.019		0.319 ± 0.060
13	0.380 ± 0.054		0.145 ± 0.051
22	0.205 ± 0.113		0.106 ± 0.035
34	0.062 ± 0.034		0.050 ± 0.030
***Soil – laboratory condition***			
1	0.742 ± 0.031		0.333 ± 0.112
5	0.600 ± 0.043		0.221 ± 0.007
13	0.326 ± 0.034		0.074 ± 0.011
22	0.252 ± 0.023		0.059 ± 0.006
34	0.048 ± 0.003		<LOQ
***Rapeseed plant***			
22	0.009 ± 0.009	0.005 ± 0.001	
34	<LOQ	<LOQ

Table 1, Figs. 1 and 2 show the average level and the dissipation curves for clomazone and metazachlor residues in soil samples collected after 1, 5, 13, 22 and 34 days, under field and laboratory conditions, respectively. Dissipation of clomazone and metazachlor in soil under field conditions follows first-order kinetics (y = 0.860e^−0.073×^, R^2^ = 0.978 for clomazone, and y = 0.461e^−0.068×^, R^2^ = 0.964 for metazachlor), with half-lives of 9.5 days for clomazone and 10.2 days for metazachlor. Under the laboratory conditions, dissipation of clomazone and metazachlor in soil also follows first-order kinetics (y = 0.915e^−0.079×^, R^2^ = 0.938 for clomazone and y = 0.424e^−0.122×^, R^2^ = 0.937 for metazachlor), with half-lives of 8.8 days for clomazone and 5.7 days for metazachlor.

Due to the slow growth of plants, first sampling of rapeseed for tests was possible on day 22 after the spraying. The average herbicide residue levels in these plants are shown in Table 1. 22 days after the treatment, the herbicide residue levels in rapeseed plants were below the MRL levels for Brassica plants (<0.02 mg/kg for metazachlor, and <0.01 mg/kg for clomazone)^38^. 34 days after application of herbicides, residues of these substances in rapeseed were below LOQs.

**Figure 3:**
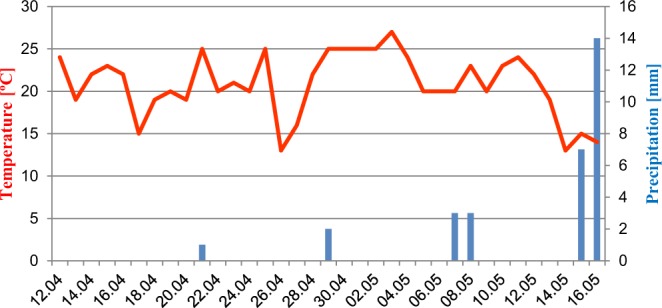
Weather conditions during the experiment.

During the experiment, a small amount of precipitation, mostly at the end of experiment, was recorded (30 mm in total during 34 days of tests) (Fig. 3).

### Confirmation of the obtained results by gas chromatography with mass spectrometry

The results obtained in field experiments were confirmed qualitatively by gas chromatography combined with mass spectrometry (GC/MS/MS). Examples of spectra for the tested compounds, rapeseed and soil samples are included in the Supplementary Fig. S2 and Fig. S3.

## Discussion

The aim of this study was to analyze dissipation of metazachlor and clomazone, herbicides widely used in rapeseed (*Brassica napus* L. subsp. *napus*) protection, applied to the soil under field and laboratory conditions. Furthermore, the uptake of these pesticides from soil by rapeseed plants was investigated under field conditions. An additional aim of this work was to modify the QuEChERS method for the determination of metazachlor and clomazone in the plant material, and validation of the method for the analyses of these substances in the soil.

In the literature, the availability of information about the dissipation of these two herbicides in soil is limited and the information about uptake of herbicide residues by rapeseed plants is scarce; therefore, these studies can be considered as a novel approach.

Before starting our research on herbicide dissipation, we validated the method for determination of these herbicides in soil, and modified and validated the QuEChERS method for herbicide determination in rapeseed. The values of characteristic parameters obtained in the validation process confirmed that the methods meet the requirements of the European Commission and are suitable for the determination of pesticide residues in soil, rapeseed, and plants with a high chlorophyll content.

Other authors used high performance liquid chromatography (HPLC) with diode array detection (DAD) for determination of clomazone in soybeans; the calculated limit of quantification for this method was 0.05 mg/kg^40^. Also in other studies in soybeans, the same technique was used using florisil as a sorbent for cleanup. In this case, the limit of quantification was 0.01 mg/kg^41^. Metazachlor was determined in oilseed rape using the reverse-phase HPLC (RP – HPLC) with UV – VIS detection, where the limit of quantification was 0.8 mg/mL^42^. In other studies, HPLC with DAD detection^41^, HPLC with UV detection, and gas chromatography with ECD detection were used to determine clomazone in soil^43^. For the determination of metazachlor in soil, gas chromatography with mass spectrometry was used, with sorbents: PSA, C18, GCB, MgSO_4_ (PSA and C18), which selectively adsorb interferences from soil, such as organic matter or humic acid (GCB adsorbs pigments and MgSO_4_ adsorbs water from the extract). In this case, the limit of quantification was 0.01 mg/kg^44^.

The aim of this study was to analyze the behavior of clomazone and metazachlor residues in soil and in rapeseed plants. Other studies showed relatively rapid dissipation of herbicides used on weeds, mainly during their germination. After application of these agents, the weeds do not grow, or after the emergence, they became white and dry out.

According to other authors, the half-life of clomazone in the soil was 7.48 days^41^ or 6–59 days^45^. Quayle *et al*.^46^ also determined the degradation time of this herbicide in soil, and it was found to be t_½_ = 14.6 days, while the maximum concentration of clomazone in the soil was 202 μg/L, therefore, after 18.4 days the compound was degraded to the levels appropriate for preservation of aquatic ecosystems. According to the Pesticide Properties Data Base (PPDB), atypical value of t_½_ for clomazone in field soil is equal to 22.6 days, but according to the EU dossier, this herbicide half-life in soil ranges between 6.3 and 145.7 days (lab studies), and 9.3 and 195 days (field studies)^47^.

On a basis of dissipation results for metazachlor in soil^48^, it can be stated that its half-life ranges between 10.92 and 12.68 days, and this herbicide was detected in the soil even 170 days after spraying. Levels of metazachlor residues in the soil were also calculated for the years 2003–2005 by Kucharski and Badowski^43^. The obtained values were within range of <0.001–0.021 mg/kg. Taking into account the results from the whole research period, at the time of crop harvesting the residues of the tested herbicide were detected in 80% of soil samples^43^. According to PPDB for soil in field conditions, a typical value of t_½_ for metazachlor in soil is equal to 8.6 days, but according to the EU dossier, half-lives for this herbicide in soil range from 5.5 to 25.3 days (lab studies) and 2.8 to 21.3 days (field studies)^47^.

The results of our study indicate that temperature can influence dissipation of clomazone and metazachlor in soil. In the laboratory conditions clomazone and metazachlor half-lives were shortened by 0.7 days and 4.5 day, respectively, in comparison to the field values. The average temperature was 20.8 °C (ranging from 13 to 27 °C) in the field and was maintained at 22 °C ± 1 °C in the laboratory conditions. The higher and stable temperature, together with the more stable moisture content of soil in the laboratory (36 ± 2%) than in the field (35 ± 10%) could lead to a more effective biodegradation process. Generally, as the temperature and humidity increase, the microbial degradation process accelerates. Periods of drought or low temperatures inhibit the growth of microorganisms, resulting in slowing down the decomposition of herbicides^11^.

According to other authors, microbial metabolisms of clomazone and metazachlor form a major degradation pathway^49,50^. The rate of clomazone degradation is affected by soil moisture, temperature, microorganisms, pH, and is accelerated under anaerobic conditions^51^. The degradation of metazachlor may be affected by soil type, soil texture fraction, organic carbon content, and pH, and is faster under anaerobic conditions^52,53^.

In the present research, the levels of herbicide residues in rapeseed plants were calculated. The herbicide residues in rapeseed plants 22 days after the treatment were below the MRLs for Brassica plants (<0.01 mg/kg for clomazone)^38^. The literature does not contain any data on the clomazone residues uptake by rapeseed. However, clomazone residues at a level of <0.01 mg/kg were found in soybeans^41^. Similar results on the clomazone dissipation, of t_½_ of 4.9 days and residue levels ranging between 0.0037 and 0.033 mg/kg, were obtained in studies on soybean plants^40^.

In the experiments concerning metazachlor conducted by Koleva-Valkova *et al*.^42^ on rapeseed leaves, the active substance was applied in two ways, into soil and foliarly. In the first case, the highest concentration (14.6 mg/kg) was recorded in the leaves 28 days after herbicide treatment. The concentration level was 4.4 mg/kg and 1.23 mg/kg after 48 and 68 days, respectively. Following the foliar application, the metazachlor residue was determined after 8 and 20 days and amounted to 4.24 mg/kg and 0.95 mg/kg, respectively. Kucharski and Badowski^43^ analyzed metazachlor uptake by white mustard (*Sinapis alba*). The maximum value of this herbicide in seeds was 0.009 mg/kg which was below MRL.

Many scientists concluded that in addition to the environmental conditions, the applied concentration and type of pesticide formulation, the physical and living state of the plant surface, the relation between the treated surface and plant weight, are the most effective factors acting on the initial deposits of the pesticides^54,55^.

## Conclusions

The modified QuEChERS-method with extracts cleanup using florisil or PSA followed by the analysis by GC-µECD, could be used with satisfying results for quantification of clomazone and metazachlor in rapeseed plants, and this method met the criteria for methods for determining pesticide residues.

The higher and stable temperature, together with the stable moisture content of soil in the laboratory could lead to more efficient biodegradation of tested herbicides.

Application of clomazone and metazachlor directly after rapeseed sowing results in a very low uptake of these substances by plants.

## Materials and Methods

### Soil properties

Soil characteristics (soil type, granulometric composition, pH, assimilable phosphorus, total carbon, organic carbon, humus content, total nitrogen, phosphorus, magnesium, potassium and calcium) were determined in laboratory of The Institute of Soil Science and Plant Cultivation in (Pulawy, Poland) accredited by The Polish Centre for Accreditation (certificate no AB 339).

### Metazachlor and clomazone analysis in soil

Analytical portions of 20 g were taken from the soil samples (weight of each sample>200 g)^56^, and weighted in a 250 mL Erlenmeyer flask. 50 mL of dichloromethane (Sigma-Aldrich Sp. z o.o., Poland) and acetone (Chempur, Poland) mixture (9:1; v/v) were added and shaken for 1 h on a GFL 3006 shaker (Germany). The extracts were allowed to stand for 10 min and then decanted through a layer of anhydrous sodium sulfate(VI) Na_2_SO_4_ (POCH, Poland) placed in a funnel. The soil samples were washed twice with 20 mL of dichloromethane, and the combined extracts were evaporated to dryness on a rotary evaporator (Heidolph Laborota 4000 Efficient, Germany) at temperature below 40 °C. The residues were then dissolved in 10 mL of petroleum ether (Chempur, Poland)^57^.

### Metazachlor and clomazone analysis in rapeseed

For the determination of clomazone and metazachlor in the plant material of a high chlorophyll content (rapeseed), the standardized method: PN-EN 15662:2018 “Foods of plant origin–Multimethod for the determination of pesticide residues using GC- and LC-based analysis following acetonitrile extraction/partitioning and clean-up by dispersive SPE–Modular QuEChERS-method” was modified^37^. The abovementioned standard does not provide guidelines for rapeseed plants regarding the amount of sample to be analyzed, the addition of water before extraction, and sorbents used for the purification of extracts. The detection technique was changed from GC-MS in the normalized method to GC-μECD.

The analytical sample of the plant material was ground in a homogenizer (Waring Commercial 8010 EG blender, USA) and mixed thoroughly, not allowing separation of the juice from the solids. An analytical portion of 5 g was taken and placed in a 50 mL centrifuge tube, then 5 mL of distilled water and 10 mL of acetonitrile (Chempur, Poland) were added. The contents of the tube were shaken for 1 min. Next, a mixture of buffer salts: 4 g MgSO_4_ (Chempur, Poland), 1 g NaCl (Chempur, Poland), 0.5 g hydrated disodium hydrogen citrate (Chempur, Poland), an 1 g hydrated trisodium citrate (Chempur, Poland),was added, and the sample was shaken again for 1 min, and centrifuged at >3000 rpm (MPW-350R, MPW MED. INSTRUMENTS, Warsaw, Poland) for 5 min. 6 mL of the acetonitrile layer was transferred to a 15 mL propylene tube containing sorbent for clean-up extracts:

(i) 150 mg Primary Secondary Amine (PSA Agilent, USA), and 900 mg MgSO_4_,
(ii) 1 g of florisil (Sigma-Aldrich Sp. z o.o., Poland) and 900 mg of MgSO_4._

Once again, the rapeseed was shaken vigorously for 1 min and then centrifuged at >3000 rpm (MPW-350R, MPW MED. INSTRUMENTS, Warsaw, Poland) for 5 min. 1 mL of extract was transferred to a 2 mL chromatographic glass vial. Acetonitrile was evaporated under a nitrogen stream and then 1 mL of petroleum ether (Chempur, Poland) was added.

### Pesticide analytical standards

Certified analytical standards of metazachlor and clomazone were purchased from Ehrenstorfer (Germany). Stock solutions of approximately 1000 μg/mL were prepared in acetone and stored at −16 °C, and were used to prepare intermediate concentration standards by dilution with acetone (stored at 4 °C). Working standard mixtures were obtained by diluting the intermediate concentration solutions with appropriate volumes of petroleum ether (stored at 4 °C).

### Chromatographic and mass spectrometry analysis

A 7890 A gas chromatograph (Agilent Technologies, Palo Alto, CA, USA) with a micro electron capture detector (GC-μECD) was used to qualify and quantify herbicide residues in soil and rapeseed samples. A mass detector equipped with three quadrupoles, model 7000 (GC-MS/MS QqQ) was used to confirm metazachlor and clomazone residues in plant and soil material. Details of chromatographic parameters are provided in Supplementary Tables S3 and S4.

In the MRM mode, multiple reactions can be monitored and selected fragmentation reactions can be observed, allowing recording the presence of relevant compounds^58^.

Clomazone and metazachlor spectra were recorded in the full scan mode and then characteristic ion fragments were selected. In the MRM mode, the following transitions were selected: 125.0 → 121.7 and 89.3 (m/z) for clomazone, and 209.1 → 132.1 and 132.1 → 117.3 (m/z) for metazachlor.

### Validation study

The validation was performed using the following parameters: trueness and precision (expressed as an average recovery and a relative standard deviation, RSD), linearity (expressed as a coefficient of determination R^2^), LOQs, and uncertainty according to the European Union guideline SANTE^35^.

The material for validation study was soil and rapeseed plants without pesticide residues. Recovery studies were used to calculate the trueness and precision of the methods. Before the analysis, samples were enriched with the standards of the pesticides active substances, at two levels: of 1 mg/kg (for both substances) and of 0.01 mg/kg for clomazone and 0.005 mg/kg for metazachlor. The samples were analyzed in five replicates (n = 5).

The LOQ for each pesticide was defined as the lowest spiking level meeting the recovery requirement and RSD for different fortification levels.

Linearity was evaluated using six-point matrix-matched standards (in “blank” matrix), of concentrations ranging 0.01–2.88 μg/mL and 0.005–2.12 μg/mL for clomazone and metazachlor, respectively.

Expanded measurement uncertainties were estimated using a “top-down” empirical model according to the data found in the validation study (coverage factor k = 2, confidence level 95%)^29,39^.

### Dissipation of herbicides in soil and rapeseed

**Figure 4:**
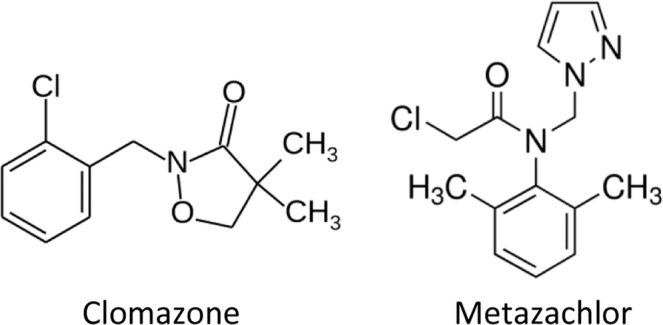
Molecular structures of herbicides.

Metax 500 SC (INNVIGO Sp. z o.o., Poland) is a preparation that combats mono-and dicotyledonous annual weeds. It is recommended in the cultivation of rapeseed and white headed cabbage. The active substance of this preparation is metazachlor (500 g of metazachlor per 1 L of the plant protection product)^59^. According to the International Union of Pure and Applied Chemistry (IUPAC) nomenclature, metazachlor is called 2-chloro-2′, 6′-dimethyl-N-(1H-pyrazol-1-ylmethyl)acetanilide (Fig. 4).

Command 480 EC (FMC Chemical s.p.r.l., Belgium) is preparation intended for combating some annual dicots and monocots in beans, peas, carrots, cucumbers, rapeseed and potatoes. The active substance of this preparation is clomazone (480 g of clomazone per 1 L of the plant protection product)^59^. According to IUPAC nomenclature, clomazone is called 2-(2-chlorobenzyl)-4, 4-dimethylisoxazolidin-3-one (Fig. 4).

The trial was conducted in an agricultural farm that implements principles of integrated production. The farm was located in Budziwój (south-eastern Poland, an administrative division of Podkarpackie Voivodeship).

On 12 April 2018, rape seeds were sown in a field of an area of 2 ha. Herbicides: Command 480 EC at a recommended dose of 0.2 L/ha, and Metax 500 SC at a dose of 1 L/ha were applied on 13 April 2018 on the whole cultivated field. The samples of soil and rapeseed were collected according to provisions of the national regulation^56^. The soil samples of 200 g were collected on five dates: 1, 5, 13, 22, and 34 days after herbicide application, with Egner’s stick (at 0-20 cm depth) following a W-shaped path across the field, in four replicates (n = 4). The plant material samples of 200 g were collected after plant emergence (on two dates), in four replicates. The rapeseed plants were very small and were analyzed as a whole. The plant material was ground in a homogenizer (Waring Commercial 8010 EG blender, USA) and mixed thoroughly, to prevent juice separation from solids. An analytical portion of 5 g was taken for herbicide analysis.

The weather conditions (the average daily temperature and precipitation) were monitored throughout the experiment^60^ (Fig. 3).

The laboratory test was conducted simultaneously with the field experiment. 400 g of soil, collected from field one day after herbicide application, were weighted into a 2 L transparent propylene container, in four replicates. Samples were stored in monitored conditions, at 22 °C ± 1 °C. Samples for pesticide analysis were taken 1, 5, 13, 22 and 34 days after treatment – on the same dates as samples from the rapeseed field. Before sampling, each soil was thoroughly mixed with a laboratory spoon.

In each soil sample, water content was measured by the weighing method after drying at 105 °C ± 3 °C (S–40, Alpina, Poland)^61^. The pesticide concentration was calculated for soil dry mass.

The dissipation kinetics of metazachlor and clomazone residues were determined by plotting the concentration against the time elapsed after treatment. It was found that they followed the exponential relationship with time, corresponding to the first-order kinetics Eq. 1:

1 $${\rm{y}}={{\rm{y}}}_{{\rm{o}}}{{\rm{e}}}^{-{\rm{kx}}}$$

where: y - concentration of the herbicide residue at a given time, y_o_ - initial herbicide concentration, k - constant rate of herbicide dissipation per day.

On the basis of this equation, the dissipation half-life periods (t_½_ = ln (2)/k) were calculated for the tested herbicides^62^.

## Supplementary information

Supplementary information.
