# Supplementary material for: The difference in dissipation of clomazone and metazachlor in soil under field and laboratory conditions and their uptake by plants
Source: Sci Rep. 2020 Feb 28;10:3747. doi: 10.1038/s41598-020-60720-0 (PMC7048729; doi:10.1038/s41598-020-60720-0)
Supplement: Supplementary file 1 — Supplementary information. [file 41598_2020_60720_MOESM1_ESM.docx]

**Supplementary information**

**The difference in dissipation of clomazone and metazachlor in soil under field and laboratory conditions and their uptake by plants**

Ewa Szpyrka, Magdalena Słowik-Borowiec, Paulina Książek, Aneta Zwolak, Magdalena Podbielska

**Table S1.** Validation parameters for soil samples: linearity, recovery and relatively standard deviation (RSD), expanded uncertainty and coefficient of determination (R^2^).

| **Active substance** | **Range of linearity**  **(µg/mL)** | **Spiking level**  **(mg/kg)** | **Average recovery (± RSD)**  **(%)** | **U (k=2)**  **(%)** | **R^2^** |
| --- | --- | --- | --- | --- | --- |
| Clomazone | 0.01 – 2.88 | 0.010 | 112 (10) | 22 | 0.998 |
|  |  | 1.000 | 96 (9) | 18 |  |
| Metazachlor | 0.005 – 2.12 | 0.005 | 88 (7) | 16 | 0.999 |
|  |  | 1.000 | 98 (13) | 27 |  |

**Table S2.** Validation parameters (recovery with relative standard deviation (RSD), correlation coefficients (R^2^) and measurement uncertainties) for metazachlor and clomazone in rapeseed plants.

| **Active substance** | **Range of linearity**  **(µg/mL)** | **Spiking level**  **(mg/kg)** | **Average recovery (± RSD)**  **(%)** | | **U (k=2)**  **(%)** | | **R^2^** |
| --- | --- | --- | --- | --- | --- | --- | --- |
|  |  |  | **Florisil** | **PSA** | **Florisil** | **PSA** |  |
| Clomazone | 0.01–2.88 | 0.010 | 106 (5) | 99 (8) | 12 | 16 | 0.998 |
|  |  | 1.000 | 109 (7) | 107 (2) | 15 | 7 |  |
| Metazachlor | 0.005–2.12 | 0.005 | 86 (12) | 93 (4) | 26 | 11 | 0.999 |
|  |  | 1.000 | 99 (2) | 101 (2) | 7 | 6 |  |

**Table S3.** Operating conditions of a chromatograph with a μECD detector.

| **Sample injection mode (volume) / temperature** | Splitless mode (2 μL) / 250 °C |
| --- | --- |
| **Capillary column** | HP-5 MS Ultra Inert/ 30 m ×0.25 mm I.D. × 0.25-μm |
| **Carrier gas / flow** | Helium (5.0purity) / 1.37 mL/min |
| **Column temperature program** | 100 °C (0 min) → 10 °C/min → 180 °C (4 min) → 3 °C/min → 220 °C  Total run time of analysis was 25.3 min |
| **Temperature of detector / gas (flow)** | 300°C/ nitrogen (5.0 purity) / 40 mL/min |
| **Software** | ChemStation, Rev. B04.03-SP2 |

**Table S4.** Operating conditions of a chromatograph with a mass detector (full scan and MRM mode).

| **Sample injection mode (volume) / temperature** | Splitless mode (1 μL) / 250 °C |
| --- | --- |
| **Capillary column** | HP-5 MS Ultra Inert/ 30 m ×0.25 mm I.D. × 0.25-μm |
| **Carrier gas / flow** | Helium (5.0 purity) / 1 mL/min |
| **Electron ionization mode** | (EI −70 eV) |
| **Temperature of the transfer line** | 250 °C |
| **Temperature of the ion source** | 230 °C |
| **Temperature of quadrupoles** | 150 °C |
| **Column temperature program** | 40 °C (2 min hold) to 220 °C at a rate 30 °C/min, increased to 260 °C at 5 °C/ min.  Total run time of analysis was 16 min |
| **Collision gases** | Helium and nitrogen |
| **Software** | Mass Hunter, version B.06.00 |


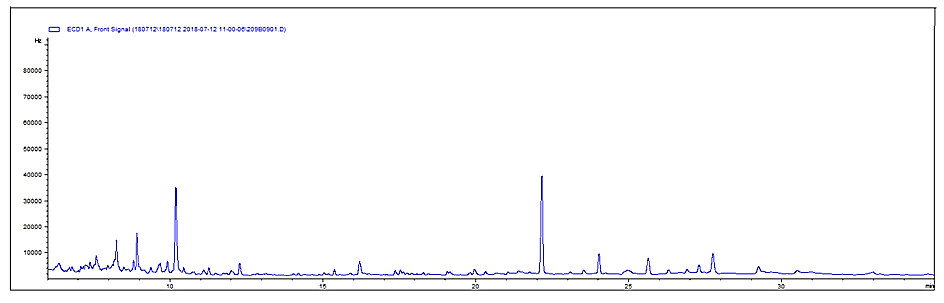


B

A


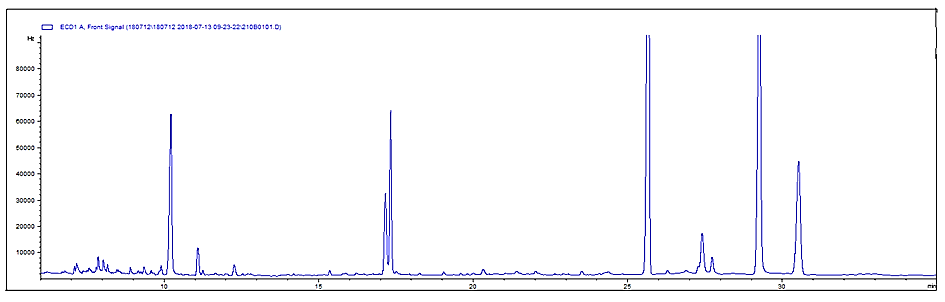


**Figure S1. µECD chromatograms of sample extracts cleanup.** A) florisil B) PSA sorbents.

A

B

**Figure S2. Full scan MS spectrum for the standard.** A) clomazone (R_t_ 10.536 min) and B) metazachlor (R_t_ 12.948 min).

A

B

**Figure S3. MRM spectrum for the rape samples and soil.** A) clomazone (transitions 125 → 121.7 and 89.3 (m/z)) and B) metazachlor (transitions 209.1 → 132.1 i 132.1 → 117.3 (m/z)).
